# *PER* Gene Family Polymorphisms in Relation to Cluster Headache and Circadian Rhythm in Sweden

**DOI:** 10.3390/brainsci11081108

**Published:** 2021-08-23

**Authors:** Felicia Jennysdotter Olofsgård, Caroline Ran, Carmen Fourier, Catalina Wirth, Christina Sjöstrand, Elisabet Waldenlind, Anna Steinberg, Andrea Carmine Belin

**Affiliations:** 1 Department of Neuroscience, Karolinska Institutet, 171 65 Stockholm, Sweden; felicia.jennysdotter.olofsgard@ki.se (F.J.O.); caroline.ran@ki.se (C.R.); carmen.fourier@ki.se (C.F.); catalina.sara.wirth@stud.ki.se (C.W.); 2 Department of Clinical Neuroscience, Karolinska Institutet, 171 65 Stockholm, Sweden; christina.sjostrand@ki.se (C.S.); elisabet.waldenlind@sll.se (E.W.); anna.steinberg@sll.se (A.S.); 3 Department of Neurology, Karolinska University Hospital, 171 64 Stockholm, Sweden

**Keywords:** pain, genetics, diurnal rhythmicity, clock genes, diurnal preference

## Abstract

The trigeminal autonomic cephalalgia, cluster headache (CH), is one of the most painful disorders known to man. One of the disorder’s most striking features is the reported diurnal rhythmicity of the attacks. For a majority of patients, the headache attacks occur at approximately the same time every day. Genetic variants of genes involved in the circadian rhythm such as Period Circadian Regulator 1, 2, and 3 (*PER1*, *2* and *3*) are hypothesized to have an effect on the rhythmicity of the attacks. Six *PER1*, *2* and *3* genetic markers; the indel rs57875989 and five single nucleotide polymorphisms (SNPs), rs2735611, rs2304672, rs934945, rs10462020, and rs228697, were genotyped, using TaqMan^®^ or regular polymerase chain reaction (PCR), in a Swedish CH case control material. Logistic regression showed no association between CH and any of the six genetic variants; rs57875989, *p* = 0.523; rs2735611, *p* = 0.416; rs2304672, *p* = 0.732; rs934945, *p* = 0.907; rs10462020, *p* = 0.726; and rs228697, *p* = 0.717. Furthermore, no difference in allele frequency was found for patients reporting diurnal rhythmicity of attacks, nor were any of the variants linked to diurnal preference. The results of this study indicate no involvement of these *PER* genetic variants in CH or diurnal phenotype in Sweden.

## 1. Introduction

Patients diagnosed with the neurological disorder cluster headache (CH) suffer from extremely painful headache attacks occurring once every other day up to eight times per day during an active cluster period [1]. Autonomic symptoms, such as lacrimation and swelling around the eye, accompany the attacks [1]. Interestingly, a majority of patients have a striking rhythmicity to their headache attacks. A study performed by our lab in 2018 showed that 66.7% of a Swedish CH cohort reported attacks appearing at certain time intervals throughout the day, with the time interval 2:00–4:00 AM being the most common time for attacks to occur [2]. Though the exact pathophysiology behind CH, and more specifically the rhythmic nature of the attacks, is yet to be fully elucidated, it is thought to have a multi-factorial cause with a genetic component.

Molecular clock proteins are found in all cells throughout the body and their expression levels control the circadian rhythm. Protein products of the genes Circadian Locomotor Output Cycles Kaput (*CLOCK*) and Brain and Muscle ARNT-Like 1 (*BMAL1*) form a heterodimer which activates the transcription of other clock genes. CLOCK/BMAL1 transcribe their own inhibitors, Period Circadian Regulator 1, 2 and 3 (*PER1/2/3*) and Cryptochrome 1/2 (*CRY1/2*). This produces a feedback loop which runs in approximately 24 h cycles and allows for the variation of expression of circadian-regulated genes throughout the day [3]. Along with this robust core genetic feedback loop, environmental cues and other circadian related genes form the complex network which dictates our diurnal rhythm.

Despite the complex nature of the circadian rhythm and the challenge to conduct large genetic studies on CH with sufficient power, several of the circadian-related genes and accompanying single nucleotide polymorphisms (SNPs) have previously been linked to CH. Our lab reported associations with rs12649507 in *CLOCK* and with rs8192440 in *CRY1* in a Swedish CH cohort [4,5]. Our lab recently published a genome wide association study (GWAS) conducted on data on over 1400 CH patients and 6000 controls [6]. This GWAS strengthened the evidence behind the role of genetics in CH and found significant loci close to genes which were indirectly involved with the circadian rhythm. One locus on chromosome 2 which reached genome wide significance overlies an intronic region of the gene MER proto-oncogene, tyrosine kinase (*MERTK*), which in turn activates the cAMP-responsive element binding protein (CREB). The CREB pathway is crucial for regulating the timing and light entrainment of the suprachiasmatic nucleus, otherwise known as the master clock [7]. The specific association previously reported between CH and the *CLOCK* and *CRY1* SNPs could not be replicated in the GWAS. However, the threshold for reaching genome wide significance could be seen as more conservative, identifying SNPs with larger effect size, than candidate gene association studies.

*PER1* and *PER2* work as light sensitive clock genes [8,9]. Interestingly, the protein pituitary adenylate cyclase-activating peptide-38 (PACAP-38), reported to have an altered expression in inter-bout CH patients, is also known to induce expression of *PER1* and *PER2*, indicating a potential link, though there is yet to be a study to test this [10,11].

In multiple studies, *PER1, 2,* and *3* variants have been associated with sleep disorders and diurnal preference. The minor allele (C) of the genetic missense variant rs2735611 in *PER1* has been linked to extreme morning preference [12]. Another SNP, rs2304672, in the *PER2* gene has been shown to be involved in circadian-related reward circuitry in the brain. The minor allele, G, was linked to reduced activity in response to emotional stimuli which in turn was associated to sleep mid-point, the midpoint between sleep onset and awakening, in adolescents, indicating it plays a role in both circadian rhythm and reward response [13]. Another SNP in *PER2*, rs934945, was associated with diurnal preference, specifically the major allele, G, which was linked to morningness [14,15]. The minor allele of rs10462020 in *PER3* has been associated with delayed sleep phase syndrome and altered morningness/eveningness score along with bipolar disorder [16,17,18]. Interestingly, there are reports on comorbidity of CH with bipolar disorder [19]. Both CH patients and bipolar disease patients can be successfully treated with the drug lithium, which is known to alter *PER3* mRNA expression [20]. The minor allele of the other *PER3* SNP, rs228697, has been found to be significantly associated with evening preference, and is more common in free-running type individuals whose circadian rhythm does not adapt to the light/dark cycle [21]. The variable tandem repeat rs57875989, with a repeat of either four or five 54 bp sequences (4/5), has been linked to diurnal preference [22], see review Archer et al. [23]. Ofte et al. studied rs57875989 in a Norwegian CH cohort to investigate if it could be linked to the diurnal rhythmicity of attacks. They did not find a difference in allele frequencies between CH patients and controls [24]. However, their cohort was relatively small (149 CH patients and 432 controls) and might not have had the power to detect a small difference. 

As the two clock genes, *CLOCK* and *CRY1*, have been implicated in CH in Sweden, we hypothesize that *PER1/2/3* variants which have been linked to circadian rhythm, could also be linked to CH risk and/or to a circadian phenotype of this disorder. We aimed to replicate the study by Ofte et al. by genotyping rs57875989 in our large Swedish CH case-control cohort. We additionally screened SNPs in *PER1* (rs2735611), *PER2* (rs2304672, rs934945) and *PER3* (rs10462020, rs228697), and stratified the results for self-reported diurnal rhythmicity of attacks as well as diurnal preference.

## 2. Materials and Methods

### 2.1. Patient Information

The patient material consisted of 524 CH patients and 680 controls (Table 1). Patients were diagnosed according to the International Classification of Headache Disorders (ICHD)–III-beta criteria [25]. CH patients were recruited from throughout Sweden in collaboration with the neurology clinic at Karolinska University Hospital. To study diurnal rhythmicity patients were asked: “At what time of day do your attacks occur?” They were considered to be positive for diurnal rhythmicity if they specified one or several two-hour intervals, while they were considered negative if they perceived their attacks as occurring at random time points. Patients who did not respond to the question were not included in the analysis (483 patients were included). The patients were also grouped based on diurnal preference (492 individuals included). Patients who consider themselves as having a diurnal preference for mornings were placed in the Morning group, while the Evening group included patients with evening as their diurnal preference. Intermediate individuals consider themselves as not belonging to either group or as part of both. The control population consisted of anonymous blood donors from the Stockholm region, representing a general Swedish population as well as 15 neurologically healthy individuals recruited at Karolinska University Hospital. Ethical permit was acquired from the Swedish Ethical Review Authority in Stockholm, Sweden (diary number 2014/656-31/4), and informed consent was acquired from each participant. The experiments were conducted in accordance with the Declaration of Helsinki adopted by the World Medical Association in regard to human tissue. DNA was extracted from blood samples according to standard procedures. 

### 2.2. Genotyping of PER1/2/3 SNPs Using qPCR

Quantitative Real-Time polymerase chain reaction (qPCR) was used to determine the allele frequency of *PER1/2/3* SNPs in CH patients and controls. qPCRs were performed according to the TaqMan^®^ SNP genotyping assay protocol with the TaqMan^®^ Genotyping MasterMix (Thermo Fischer Scientific, Waltham, MA, USA) and 5 ng DNA. Half of the recommended amount of assay was used for each plate since it was sufficient for the correct detection of all genotypes. The following assays were used: C__16285994_30 (rs2735611), C___2129919_40 (rs2304672), C___8740718_20 (rs934945), C__25956444_30 (rs10462020), and C_____10224_10 (rs228697). The qPCR was performed using the 7500 Fast Real-Time PCR system (Applied Biosystems, Foster City, CA, USA). The qPCR cycler program used was as follows; pre-PCR read 60 °C for 1 min, 95 °C for 10 min, 42 cycles of 95 °C 15 s and 60 °C 1 min, post-PCR read 60 °C for 1 min. The qPCR cycler program used for rs228697 was extended to 45 cycles. Post-PCR read for allelic discrimination was performed using the 7500 software version 2.0.4 (Applied Biosystems) supplied with the instrument.

### 2.3. Genotyping of PER3 Long Tandem Repeats Using PCR

Polymerase chain reaction (PCR) and DNA gel electrophoresis was used to genotype a *PER3* tandem long repeat variation (rs57875989) in patients and control subjects. *PER3* primers were designed with the web-based software Primer3 [26,27] (F: 5′-AAAGTGTCTTTTCATGTGCCCT-3′; R: 5′- GTGAATGTCTGGCATTGGAGTT-3′). Primer secondary structure and specificity was verified using mFOLD [28] and the NIH web-based software Primer-BLAST [29]. Primers were ordered from Thermo Fisher Scientific (Waltham, MA, USA). 24 µL Mastermix was added to 10 ng DNA (1 µL). The mastermix was composed of 0.5 mM dNTPs (Sigma, Saint Louis, MO, USA), 0.28 µM forward and reverse primers, 10× DyNAzyme MasterMix buffer (Thermo Fisher Scientific), and 1 U Taq Polymerase (Thermo Fisher Scientific) diluted in RNAse-free H_2_O.

The PCR reaction was run on a PTC-200 Peltier Thermal Cycler (Conquer Scientific, San Diego, CA, USA) with a touchdown protocol using the following cycling conditions; 95 °C for 3 min, 95 °C for 20 s, 65 °C for 20 s reduced by 0.5 °C every cycle, 70 °C for 30 s, repeat 20×, then 25 cycles of 95 °C for 20 s, 55 °C for 30 s and 70 °C for 30 s, final extension step at 72 °C for 5 min.

The PCR products (10 uL) were run on a 3.5% agarose gel at 70 V for 150 min using a BioRad PowerPac electrophoresis power supply (Thermo Fisher Scientific). A 100 bp GeneRuler DNA ladder (Thermo Fisher Scientific) was used as a reference. The *PER3* five repeat allele was identified as a 366 bp band while the *PER3* four repeat allele as a 312 bp band. The gel was then read using ImageQuant LAS 4000 (GE Healthcare, Chicago, IL, USA).

### 2.4. Statistics

Logistic regression with sex as a covariate was performed to determine if there was a significant association between CH and allele frequency, independent of whether the CH patients reported diurnal rhythmicity or not. Statistical analysis for data stratified by diurnal rhythmicity of attacks or diurnal preference was performed using a two-tailed chi-squared (χ^2^) test or a Fisher’s exact test with GraphPad Prism 6 software (GraphPad Softwares Inc., La Jolla, CA, USA). Hardy-Weinberg equilibrium (HWE), and logistic regression calculations were conducted using PLINK 1.90 [30]. A two tailed *p*-value of 0.05 was deemed significant. The PS Power and Sample Size Calculation program version 3.0 was used for power analysis [31]. The minor allele frequencies (MAFs) of rs2735611, rs2304672, rs934945, rs10462020, and rs228697 were taken from the 1000 Genomes Project Phase 3 and the MAF of rs57875989 was taken from the control population of Ofte et al. [24]. With this sample size of 524 CH patients and 680 controls, and a minor allele frequency between 0.094–0.197 based on individuals with European descent [32], we have 80% power to detect true odds ratios (OR) for CH below 0.498–0.693 or above 1.41–1.696, depending on the SNP.

## 3. Results

The SNPs were genotyped in 524 CH patients and 680 controls (Table 1). A call rate of 99.50% was achieved for rs2735611, 99.92% for rs2304672, 98.42% for rs934945, 97.8% for rs10462020, and 98.2% for rs228697. rs934945, rs57875989, rs2304672, rs10462020, and rs228697 were in accordance with HWE (*p* > 0.05), data available upon request. The *PER1* SNP rs2735611 was not in HWE for the controls (*p* = 0.023). In order to account for the higher percentage of males in the patient cohort compared to controls (65.8% vs. 56.8%), we analyzed the data using a logistic regression under an additive model with sex as a covariate. This analysis did not generate any significant association with CH for any of the SNPs; rs2735611 (*p* = 0.416), rs2304672 (*p* = 0.732), rs934945 (*p* = 0.907), rs10462020 (*p* = 0.726), rs228697 (*p* = 0.717) (Table 2).

The long tandem repeat variant rs57875989 in *PER3* was also genotyped in 524 CH patients and 680 controls using PCR (Table 1). It had a call rate of 90.2%. There was no significant association between rs57875989 and CH according to the logistic regression (*p* = 0.523) (Table 2).

Due to the role *PER* genes play in regulating the circadian rhythm, patients were stratified for self-reported diurnal rhythmicity of their attacks. There was no significant difference in allele distribution between controls, CH patients reporting diurnal rhythmicity, and CH patients not reporting rhythmicity in rs2735611 (*p* = 0.696), rs2304672 (*p* = 0.972), rs934945 (*p* = 0.750), rs10462020 (*p* = 0.826), rs228697 (*p* = 0.712), or rs57875989 (*p* = 0.384) (Table 3). 

Since these genetic variants had previously been linked to diurnal preference/chronotype, allele frequencies were also compared based on diurnal preference. We did not have diurnal preference data for controls, therefore only CH patients were included in this analysis. Patients who did not answer questions regarding diurnal preference were excluded from the analysis (492 patients were included). Patients had categorized themselves as being either morning, evening, or intermediate types. Statistical analysis showed no significant difference in allele distribution for any of the genetic variants in relation to chronotype (Table 4).

## 4. Discussion

There was no significant association found between the *PER1/2/3* genetic variants investigated and CH in our large Swedish CH cohort of over 500 patients and 600 controls. The allelic distribution of rs57875989 was similar to what was reported in a Norwegian CH cohort, as well as in other control populations, and our results confirm the lack of association between *PER3* and CH suggested by Ofte et al. [24,33,34].

To our knowledge, this is the first hypothetically-driven study investigating the association between rs2735611 (*PER1*), rs2304672, rs934945 (*PER2*), rs10462020, rs228697 (*PER3*) and CH. Our study indicates no association between these SNPs and the disease, even when the patients were sub grouped based on circadian phenotype. Though no clear link was found in this study, we cannot rule out the possibility of an association in other populations, nor that there are other *PER1/2/3* genetic variants which play a role in CH pathophysiology. We noted that the *PER1* SNP rs2735611 was not in HWE in the control population which warrants caution in interpreting our results regarding this variant. Genotyping of this variant was carefully verified and validated by rerunning samples with spurious allelic distribution to exclude methodological errors. Further, the majority of the study population consists of anonymous blood donors and therefore recruitment bias is unlikely. However, the material is not large, and we cannot completely rule out occurrence of some genetic drift.

Genotype based on diurnal preference was also investigated to see if our results could back-up previous findings by other research groups [12,14,17,21,22,34,35,36]. No significant association was found between any of the genetic variants and diurnal preference. It should be noted that only CH patients were used in this analysis since we did not have diurnal preference data for controls, and the results were based on self-reported chronotype, which is less robust than a validated morningness-eveningness questionnaire. *PER1* SNP rs2735611 was previously linked to extreme evening preference and since we did not focus on the extremities of diurnal preference this could account for the non-significance [12]. The *PER2* SNP rs2304672 was not directly linked to diurnal preference but more to the reward circuitry connected to circadian rhythm, and might not be detectable in the type of study we conducted [13]. Hida et al. found no association between rs2304672 and diurnal preference in agreement with our results [21]. The major allele of the *PER2* SNP rs934945 was more common in individuals with morning preference, in concurrence with results reported by Lee et al. and Song et al., however, the allele frequency distributions were just short of reaching statistical significance [14,15]. Johansson et al. had previously reported rs10462020 to be linked to diurnal preference in a group of seasonal affective disorder (SAD) patients and controls [17]. It is possible that CH as a disease could skew the results, although the same could be true for SAD. Hida et al. found the G allele of rs228697 to be linked with eveningness, while the C allele was connected to morningness, in a large material consisting of 925 controls [21]. There have been conflicting results regarding the long tandem repeat rs57875989 and its association with diurnal preference [22,34,35,36]. There are multiple studies which, in agreement with our results, did not find a correlation between chronotype and rs57875989 [34,35,36].

## 5. Conclusions

To conclude, no association was found in a Swedish cohort between the allelic distributions of the *PER1/2/3* variants studied and CH. There was no clear link found when stratified for diurnal rhythmicity of headache attacks. The apparent rhythmicity of CH attacks indicates a role for biological clock genes, and though no connection was found for these *PER1/2/3* variants, further investigation is needed to clarify the potential part of the *PER* genes in the pathophysiology of CH.

## Figures and Tables

**Table 1:** Demographic information.

	CH Patients	Controls
Number of individuals	524	680
Age (years)	51.8	*n*/a
Age at onset (years)	32.6	*n*/a
Male % (*n*)	65.8 (345)	56.8 (386)
Heredity % (*n*) *	10.0 (47)	*n*/a
Diurnal Rhythmicity % (*n*) **	69.8 (337)	*n*/a

CH = cluster headache, *n* = number of individuals, Heredity = patients reporting having one or more first-, second- or third-degree relative(s) with CH, * Based on 472 individuals for whom this information was available ** Based on 483 individuals for whom this information was available.

**Table 2:** Allele frequencies of genetic variants in *PER1*, *PER2*, and *PER3* in cluster headache patients vs controls.

		Allele	Allele Frequency		OR (95% CI)	*p*-Value
			CH Patients % (*n*)	Control % (*n*)		
*PER1*	rs2735611	A	85.0 (889)	86.2 (1164)	1.10	0.416
		G	15.0 (157)	13.8 (186)	(0.88–1.37)	
*PER2*	rs2304672	G	88.3 (925)	87.8 (1192)	0.96	0.732
		C	11.7 (123)	12.2 (166)	(0.75–1.22)	
	rs934945	C	80.7 (831)	81.1 (1087)	1.01	0.907
		T	19.3 (199)	18.9 (253)	(0.82–1.24)	
*PER3*	rs10462020	T	80.8 (835)	81.0 (1071)	1.06	0.726
		G	19.3 (199)	19.0 (251)	(0.78–1.43)	
	rs228697	C	89.9 (928)	89.9 (1198)	0.91	0.717
		G	10.1 (104)	10.1 (134)	(0.54–1.52)	
	rs57875989	4′	66.4 (667)	66.3 (774)	0.93	0.523
		5′	33.6 (337)	33.7 (394)	(0.75–1.16)	

CH = cluster headache, *n* = number of alleles, OR = odds ratio, 95% CI = confidence interval of 95%, 4′ = rs57875989 allele with 4 repeats, 5′ = rs57875989 allele with 5 repeats. OR and *p*-values are based on a logistic regression using sex as a covariate.

**Table 3:** Diurnal rhythmicity of attacks in relation to allele frequency of *PER1*, *PER2*, and *PER3* genetic variants in cluster headache patients.

		Allele	Allele Frequency			χ^2^ (df)	*p*-Value
			CH Patients		Control % (n)		
			Diurnal Rhythmicity % (*n*) *	No Rhythmicity % (*n*) *			
*PER1*	rs2735611	A	84.8 (570)	85.6 (250)	86.2 (1164)	0.72 (2)	0.696
		G	15.2 (102)	14.4 (42)	13.8 (186)		
*PER2*	rs2304672	G	88.1 (594)	88.0 (257)	87.8 (1192)	0.06 (2)	0.972
		C	11.9 (80)	12.0 (35)	12.2 (166)		
	rs934945	C	81.7 (544)	79.6 (226)	81.1 (1087)	0.58 (2)	0.750
		T	18.3 (122)	20.4 (58)	18.9 (253)		
*PER3*	rs10462020	T	80.9 (542)	79.4 (224)	81.0 (1071)	0.38 (2)	0.826
		G	19.1 (128)	20.6 (58)	19.0 (251)		
	rs228697	C	90.4 (600)	88.6 (257)	89.9 (1198)	0.68 (2)	0.712
		G	9.6 (64)	11.4 (33)	10.1 (134)		
	rs57875989	4’	65.2 (428)	69.9 (197)	66.3 (774)	1.91 (2)	0.384
		5’	34.8 (228)	30.1 (85)	33.7 (394)		

CH = cluster headache, *n* = number of alleles, χ^2^ = chi-square test, df = degrees of freedom, 4’ = rs57875989 allele with 4 repeats, 5’ = rs57875989 allele with 5 repeats. * Based on 483 individuals for whom this information was available.

**Table 4:** Allele frequency of *PER1*, *PER2*, and *PER3* genetic variants in relation to diurnal preference in cluster headache patients.

		Allele		Allele Frequency		χ^2^ (df)	*p*-Value
			Morning% (*n*) *	Intermediate% (*n*) *	Evening% (*n*) *		
*PER1*	rs2735611	A	86.5 (289)	85.3 (232)	84.0 (316)	0.87 (2)	0.648
		G	13.5 (45)	14.7 (40)	16.0 (60)		
*PER2*	rs2304672	G	87.2 (293)	90.4 (246)	86.4 (325)	2.54 (2)	0.281
		C	12.8 (43)	9.6 (26)	13.6 (51)		
	rs934945	C	84.3 (280)	76.9 (203)	80.9 (301)	5.30 (2)	0.071
		T	15.7 (52)	23.1 (61)	19.1 (71)		
*PER3*	rs10462020	T	77.3 (258)	82.0 (218)	82.7 (306)	3.77 (2)	0.152
		G	22.8 (76)	18.1 (48)	17.3 (64)		
	rs228697	C	91.5 (300)	87.7 (235)	90.4 (338)	2.43 (2)	0.296
		G	8.5 (28)	12.3 (33)	9.6 (36)		
	rs57875989	4′	67.8 (221)	65.2 (172)	66.0 (239)	0.49 (2)	0.783
		5′	32.2 (105)	34.9 (92)	34.0 (123)		

*n* = number of alleles, χ^2^ = chi-square test, df = degrees of freedom, 4′ = rs57875989 allele with 4 repeats, 5′ = rs57875989 allele with 5 repeats. * Based on 492 individuals for whom this information was available.

## Data Availability

The data presented in this study are available on request from the corresponding author. The data are not publicly available due to privacy restrictions.
